# Identification of Immune-Related Key Genes in Ovarian Cancer Based on WGCNA

**DOI:** 10.3389/fgene.2021.760225

**Published:** 2021-11-15

**Authors:** Qingli Quan, Xinxin Xiong, Shanyun Wu, Meixing Yu

**Affiliations:** ^1^ Guangzhou Women and Children’s Medical Center, Guangzhou Medical University, Guangzhou, China; ^2^ School of Life Sciences, Fudan University, Shanghai, China; ^3^ Department of Biology, Faculty of Science, University of British Columbia, Vancouver, BC, Canada

**Keywords:** ovarian cancer, prognostic biomarkers, immune microenvironment, immune cells infiltration, WGCNA, CIBERSORT

## Abstract

**Background:** Ovarian cancer (OV) is a fatal gynecologic malignancy and has poor survival rate in women over the age of forty. In our study, we aimed to identify genes related to immune microenvironment regulations and explore genes associated with OV prognosis.

**Methods:** The RNA-seq data of GDC TCGA Ovarian Cancer cohort of 376 patients was retrieved from website. Weighted gene co-expression network analysis (WGCNA) and ESTIMATE algorithm were applied to identify the key genes associated with the immune scores. The correlation between key genes and 22 immune cell types were estimated by using CIBERSORT algorithms.

**Results:** WGCNA showed that the pink module was most correlated with the immune score. Seven of 14 key genes (FCRL3, IFNG, KCNA3, LY9, PLA2G2D, THEMIS, and TRAT1) were significantly associated with the OS of OV patients. Correlation analysis showed our key genes positively related to M1 macrophages, CD8 T cells, plasma cells, regulatory T (Treg) cells and activated memory CD4 T cells, and negatively related to naive CD4 T cells, M0 macrophages, activated dendritic cells (DCs) and memory B cells. Kaplan-Meier survival analysis showed that lower abundances of neutrophils and higher abundances of M1 macrophages, plasma cells, T cells gamma delta (γδT) cells and follicular helper T (Tfh) cells predicted better OV prognosis.

**Conclusion:** Forteen key genes related to the immune infiltrating of OV were identified, and seven of them were significantly related to prognosis. These key genes have potential roles in tumor infiltrating immune cells differentiation and proliferation. This study provided potential prognostic markers and immunotherapy targets for OV.

## Introduction

Ovarian cancer (OV), a fatal gynecologic malignancy, is the second most common gynecologic in women over the age of forty (Vargas, 2014). In 2018, a total of 295,414 new cases of OV was reported, with 184,799 deaths among them (Gaona-Luviano et al., 2020). OV can be categorized into three main types: epithelial, germ cell, and sex-cord-stromal. Epithelial ovarian cancer is the most common type, accounting for over 90% of total OV (Jayson et al., 2014). 70–80% of epithelial ovarian cancer subtype is high-grade serous carcinomas(Stewart et al., 2019), which is prone to peritoneal metastasis early and chemotherapy resistance(Christie and Bowtell, 2017).

Because of its generally vague symptoms such as abdominal pain and bloating, over 70% of OV are diagnosed when the disease has already progressed to stage III or IV (Stewart et al., 2019), making patients hard to receive timely treatment, leading to a poor the survival rate of patients with OV(Goff et al., 2004). The 5-year survival for OV patients is 46.2% (Chien and Poole, 2017). The over-all survival (OS) rates significantly decrease over the time. In the United Kingdom, the average OS rate for 1 year, 5 and 10 years are 71.7, 41.6, and 36.3%, respectively (Research, m2017). Additionally, OS rate remarkably correlated with OV stage. Five-year OS rate of OV is 67.7% at stage II, however, it dropped to 26.9 and 13.4% at stage III and IV, respectively (Research, 2017). The traditional treatment of OV is surgery followed by combination chemotherapy, as much as cancer tissue should be removed (Pearce et al., 2012). However, OV patients tend to have poor prognosis. Hence, the discovery of practical prognostic markers is an urgent need for OV clinical diagnosis and treatment.

Several independent studies verified that immune cell infiltration affects the courses and therapeutic effects of OV (Josephs et al., 2017; Jelinic et al., 2018). The ovarian tumors usually been infiltrated by activated T cells before being diagnosed (Nelson, 2008). What is more, patients with dense infiltrates of CD3+/CD8+ T cells associated with more favorable clinical outcomes, indicating that host immunity may plays critical role in delaying or preventing tumor recurrence after standard treatments (Nelson, 2008). Hence, finding the factors that regulate immune cell infiltration will provides us a better understanding OV (Goode et al., 2017).

In this study, the immune score of each sample was calculated by “*ESTIMATE*” R package (Yoshihara et al., 2013) and the relative abundances of various subtypes of tumor-infiltrating immune cells were defined by CIBERSORT algorithms (Chen et al., 2018) based on the RNA-seq data. The immune scores were used as a phenotype in weighted gene co-expression network analysis (WGCNA) (Langfelder and Horvath, 2008). Furthermore, we analyzed the key genes and the abundance of immune cells to reach the conclusion that increasing immune response while appropriately increasing immune regulation is beneficial to the prognosis of OV. Key genes strongly associated with immune infiltration and OV prognosis were identified. Our findings will provide a better understanding of prognostic signatures and immune therapeutic targets for OV.

## Materials and Methods

### Acquiring Data and Calculating Immune Scores

Through searching on databases (https://gdc.xenahubs.net), the RNA-seq data of GDC TCGA Ovarian Cancer cohort of 379 patients were downloaded (Dataset ID: TCGA-OV.htseq_fpkm.tsv). After filtering out 3 samples with poor quality sequencing data or lacking of phenotype massages, 376 samples were retained. Data from different datasets was averaged and combined into genomicMatrix. Then all data was log_2_(x+1) transformed and prepossessed by transforming to official gene symbols via gencode.v22.annotation.gene.probeMap. The corresponding clinical information (including diseases type, age, grade, stage and survival data) (Supplementary Table S1) were acquired from the above website. The ESTIMATE algorithm (“*ESTIMATE*” package in R software) was incorporated to calculate the immune score of each tumor sample (Yoshihara et al., 2013).

### Weighted Gene Co-Expression Network Analysis

WGCNA utilizes a systematic approach to cluster number of genes that have similar expression patterns, transforming the expression of genes into modules (Langfelder and Horvath, 2008). In our study, a coefficient of variable (CV, CV = Standard Deviation/Mean) based on 376 samples was used as a criterion for genes screening. Then, 5,098 genes with CV values greater than one were chosen for WGCNA. The “*WGCNA*” package in R software was applied to construct and visualize the network. The network development processes were conducted with a soft thresholding parameter (*β*) determined by the lowest power achieved when the scale free topology model fit was 0.9. A cluster dendrogram was drawn to visualize the modules represented in different colors while the dynamic modules with high similarities were combined into one at the cutline of 0.2. To identify the module of the strongest association with immune score, the Pearson correlation analysis was conducted to examine the relationship among gene modules. The gene significance (GS) and module membership (MM) values were also generated. The key immune-related genes were selected when the GS > 0.5 and MM > 0.7.

### Function Enrichment Analysis

Function Enrichment Analysis was performed by using the “*clusterProfiler*” R package (Yu et al., 2012). The Gene Ontology (GO) and Kyoto Encyclopedia of Genes and Genomes (KEGG) analysis was conducted for the exploration of the involved biological functions and pathways of the above 14 genes. p.adjust value less than 0.05 was defined as the criteria of significant terms.

### Comparation of Key Genes in the GEPIA Database

Gene expression profiling interactive analysis (GEPIA) is a web tool to provides key interactive and customizable functions based on tremendous amount of RNA sequencing in TCGA and GTEx databases (Tang et al., 2017). Here, we applied the GEPIA to compare the expression levels of 14 key genes between normal samples (*n* = 88) and OV samples (*n* = 426). The default parameters were *p*-value Cutoff of 0.05 and log_2_ (TPM +1).

### Analysis of Clinical Characteristics of OV and Correlation Analysis of Key Genes

The associations between the 14 key genes and clinical characteristics of OV, including diseases type, age, grade, stage and survival data, were further analyzed. Then, the correlation matrix of these genes was plotted.

### Key Genes Associate With Prognosis

To evaluate the ability of key genes in the prediction of the prognosis of OV patients, 376 patients were divided into low and high groups according to the median expression level of key genes, Then, a survival curve was drawn based on the OS status and OS.time information of each sample by Kaplan-Meier survival analysis by use of R package “*survival*” (Huang et al., 2020). The log-rank test was carried out to measure the statistical significance (Huang et al., 2020). The log-rank test was carried out to measure the statistical significance.

### Immune Cells Profile

CIBERSORT was applied to estimate the relative abundances of immune cell types through gene expression profilings which further implied about the tumor immune filtration levels of OV (Chen et al., 2018). In addition, the analyses of the relationship between the expressions of key genes and relative proportions of immune cell types including macrophages M1, B cells naive, T cells CD4 memory activated, Dendritic cells resting, plasma cells, T cells CD8, T cells regulatory (Tregs), Dendritic cells activated, B cells memory, T cells CD4 naive, monocytes, macrophages M0, mast cells resting, eosinophils, natural killer (NK) cells activated, T cells follicular helper (Tfh), T cells gamma delta (γδT), T cells CD4 memory resting, neutrophils, mast cells activated, NK cells resting, macrophages M2 were performed using the “*corrplot*” R package.

### Relative Abundances of Immune Cells Associated With Prognosis

To evaluate the prognostic ability of the relative abundances of immune cells, 376 patients were divided into low and high groups according to the relative abundances of each immune cell subtype, Then, a survival curve was drawn based on the OS status and OS.time information of each sample by Kaplan-Meier survival analysis. The log-rank test was carried out to measure the statistical significance. The log-rank test was carried out to measure the statistical significance.

### Statistical Analyses

All the statistical analyses in this study were conducted in the R 4.0.3 environment. The log-rank test was employed to compare the survival rates among the high- and low-expression groups. In the GDC TCGA database, the differential analysis method for key genes with clinical characteristics (disease type, grade, stage and sample type) was Kruskal-Wallis. In the GEPIA database, the differential analysis method for key genes was one-way analysis of variance (ANOVA) which provided by its website (http://gepia.cancer-pku.cn), using disease state (tumor or normal) as variable for calculating differential expression. Differences were significant when *p* < 0.05.

## Results

### Construction of Co-Expression Network in OV

**FIGURE 1:**
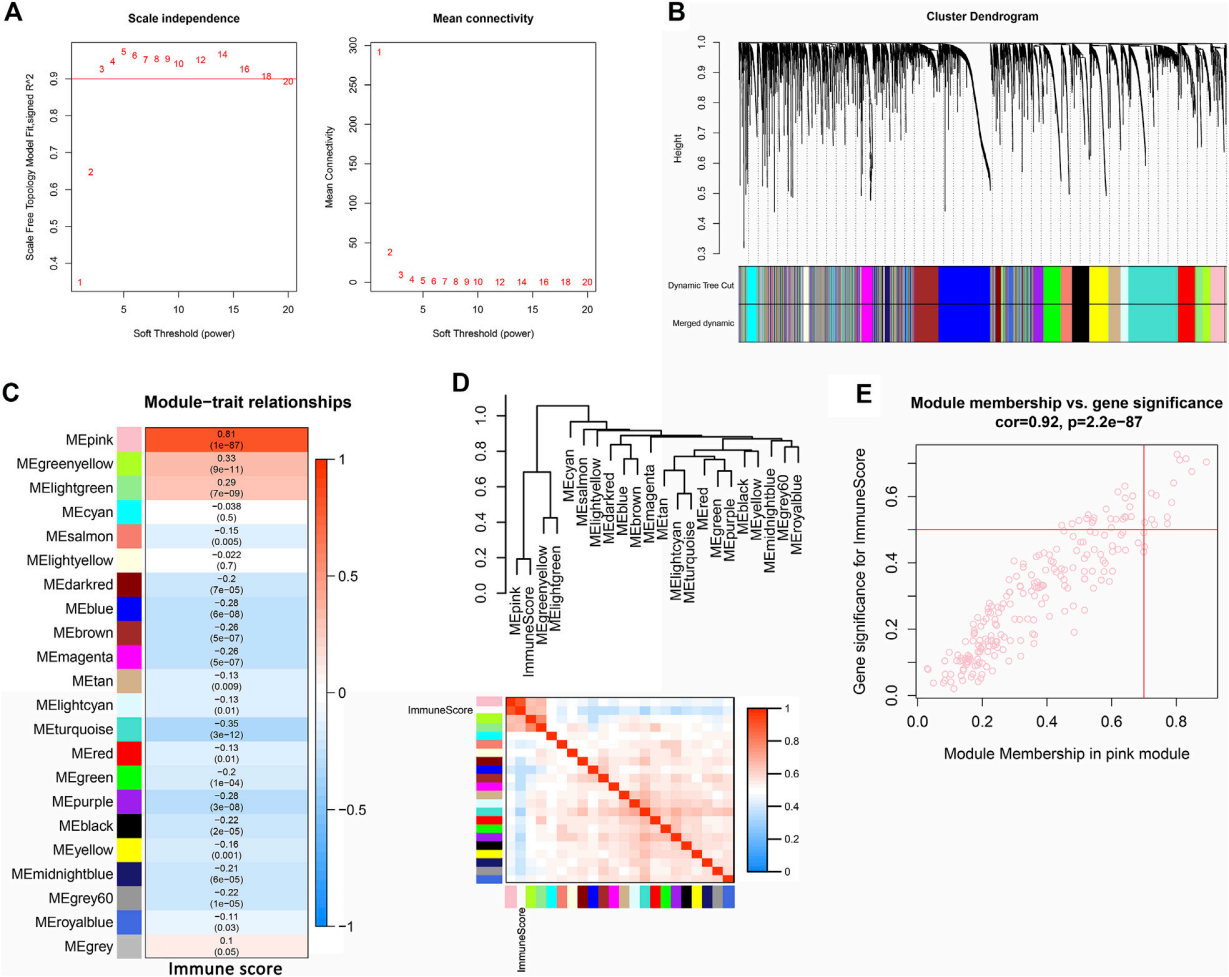
Identification of immune-related module and key genes of OV. **(A)** Graphs of scale independence, mean connectivity and scale-free topology, the appropriate soft-power was 3. **(B)** Cluster dendrogram of the co-expression network modules (1-TOM) **(C)** Analysis of correlations between the modules and immune scores, *p*-values are shown. **(D)** Cluster plot analysis of the relationship between immune scores and modules. **(E)** Scatter plot analysis of the pink module. Key genes were screened out in the upper-right area where GS >0.5 and MM >0.7 (TOM, topological overlap matrix. GS, gene significance. MM, module membership).

WGCNA was used to analyze the expression values of 5,098 genes in 376 samples. The soft-thresholding power was three which was determined based on a scale-free *R* ^2^ (*R* ^2^ = 0.9) (Figure 1A). Twenty-two modules were identified when the DissThres was set as 0.2 after merging dynamic modules, as shown in the clustering dendrograms (Figure 1B). The pink module exhibited the strongest correlation with immune scores (Figure 1C) (PCC = 0.81, p = 1E-87). The Figure 1D also indicated that the pink module was most correlated to the immune score. Finally, 14 key genes, including IFNG, FASLG, TRAT1, THEMIS, LY9, FCRL3, LAX1, IRF4, CCR2, KCNA3, IL9R, KLRC4-KLRK1, PLA2G2D, and TIFAB, were screened out for the subsequent analysis by setting the thresholds of GS > 0.5 and MM > 0.7 (Figure 1E).

### GO and KEGG Analysis of Key Genes

**FIGURE 2:**
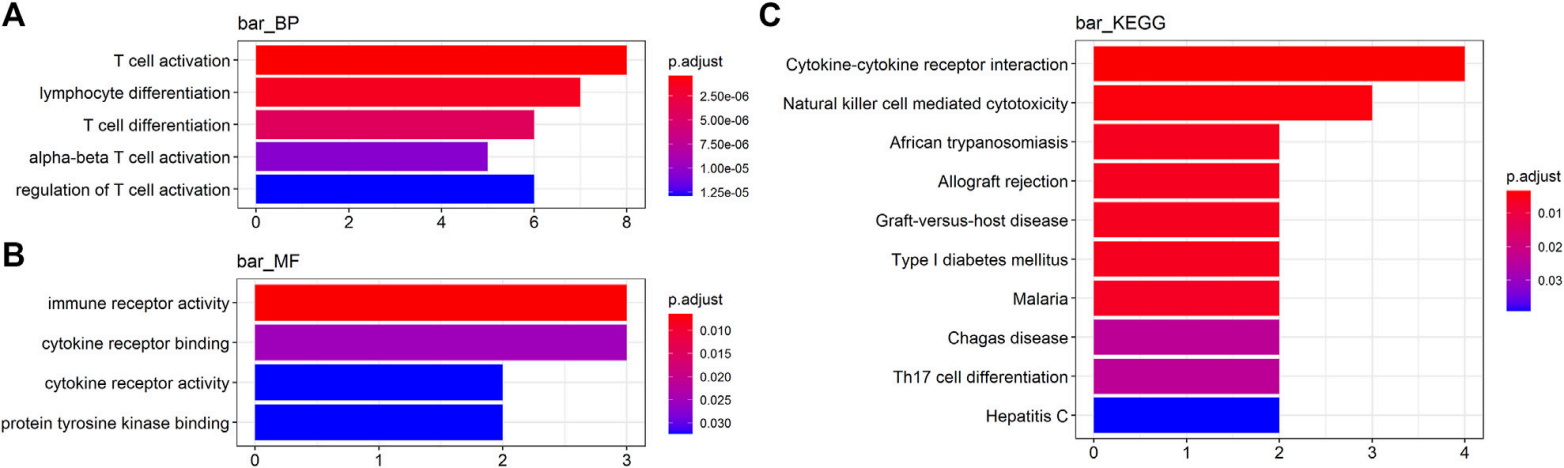
Functional analyses of the key genes. **(A, B)** Gene Ontology (GO) analysis and **(C)** Kyoto Encyclopedia of Genes and Genomes (KEGG) analysis (BP, biological process. MF, molecular function).

To obtain a deeper insight into the functions of these key genes, GO annotation and KEGG pathway enrichment analyses were employed (Supplementary Table S2). GO biological process (BP) analysis revealed that IFNG, PLA2G2D, CCR2, LAX1, LY9, IRF4, THEMIS, and KLRC4-KLRK1 were markedly enriched in T cell activation and differentiation (Figure 2A). For GO molecular function (MF) analysis, CCR2, IL9R, KLRC4-KLRK1, IFNG, and FASLG were enriched in immune receptor activity and binding (Figure 2B). KEGG analysis indicated that IFNG, FASLG, CCR2, IL9R, and KLRC4-KLRK1 were enriched in cytokine-cytokine receptor interaction and NK cell mediated cytotoxicity (Figure 2C).

### Comparison of the Expression Levels of Key Genes Between OV Samples and Normal Samples in the GEPIA Database

**FIGURE 3:**
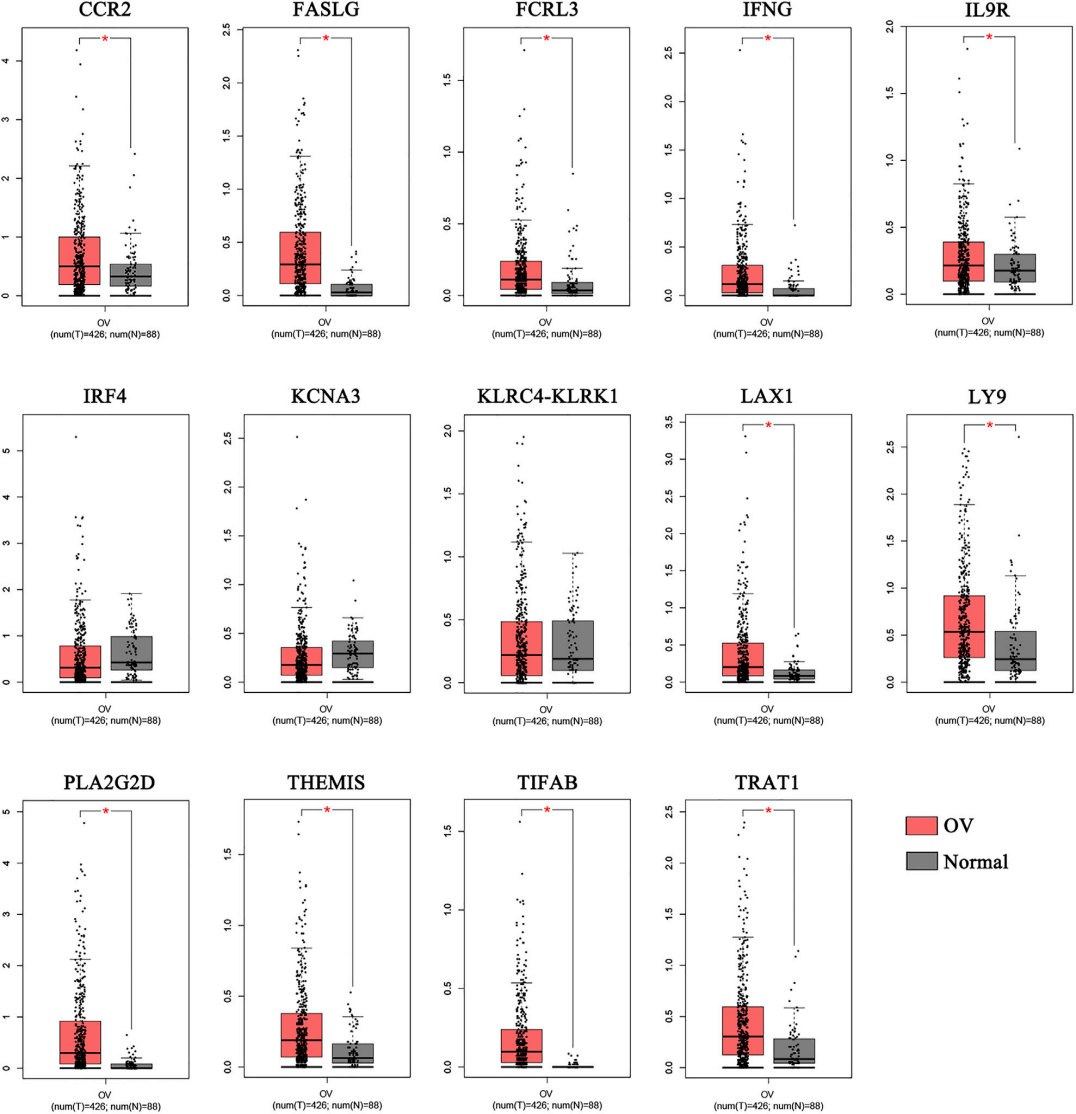
The comparison of the expressions of the key genes between the normal and OV samples. The comparison of the expressions of the key genes between the normal and OV samples. (*p < 0.05; OV, Ovarian cancer).

We compared the 14 key genes in the GEPIA database.Results indicated that the expression levels of 11 of the 14 key genes, including CCR2, FASLG, FCRL3, IFNG, IL9R, LAX1, LY9, PLA2G2D, THEMIS, TIFAB, and TRAT1,were higher expressed in OV samples compared with normal samples (Figure 3).

### Associations With Clinical Characteristics and Correlation Analysis of Key Genes

**FIGURE 4:**
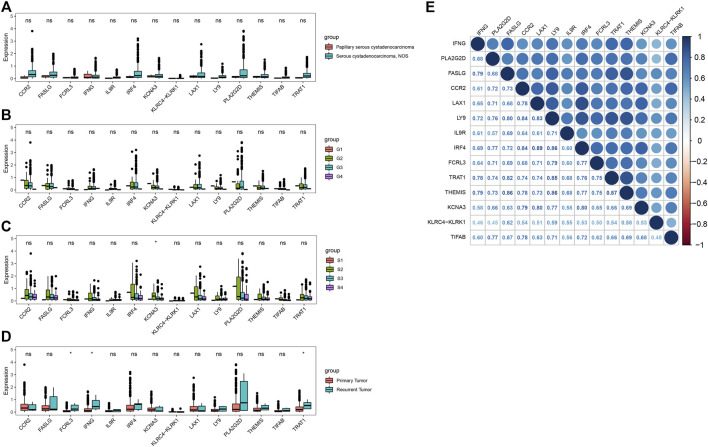
Correlation analysis of the key genes in OV. **(A–D)** Correlations of key genes with disease type, grade, stage and sample type. **(E)** Correlation analysis between key genes (*p < 0.05; ns, no significance).

We next explored the associations between the 14 key genes and clinical characteristics, including disease type, grade, stage and sample type. The expression of all 14 key genes had no statistical difference with the different grade and different disease type (Figures 4A,B). The expression levels of KCNA3 decreased with increasing tumor grade in stage Ⅱ,Ⅲ and Ⅳ (Figure 4C). What is more, there were higher expressions of FCRL3, IFNG, and TRAT1 in recurrent tumor(*n* = 5) compared with primary tumor(*n* = 371) (Figure 4D). Moreover, the Pearson correlation analysis indicated that there were significantly positively correlations between the 14 key genes (Figure 4E). The minimum and the maximum correlation coefficients were 0.45 and the was 0.89 respectively.

### Key Genes Associate With Prognosis

**FIGURE 5:**
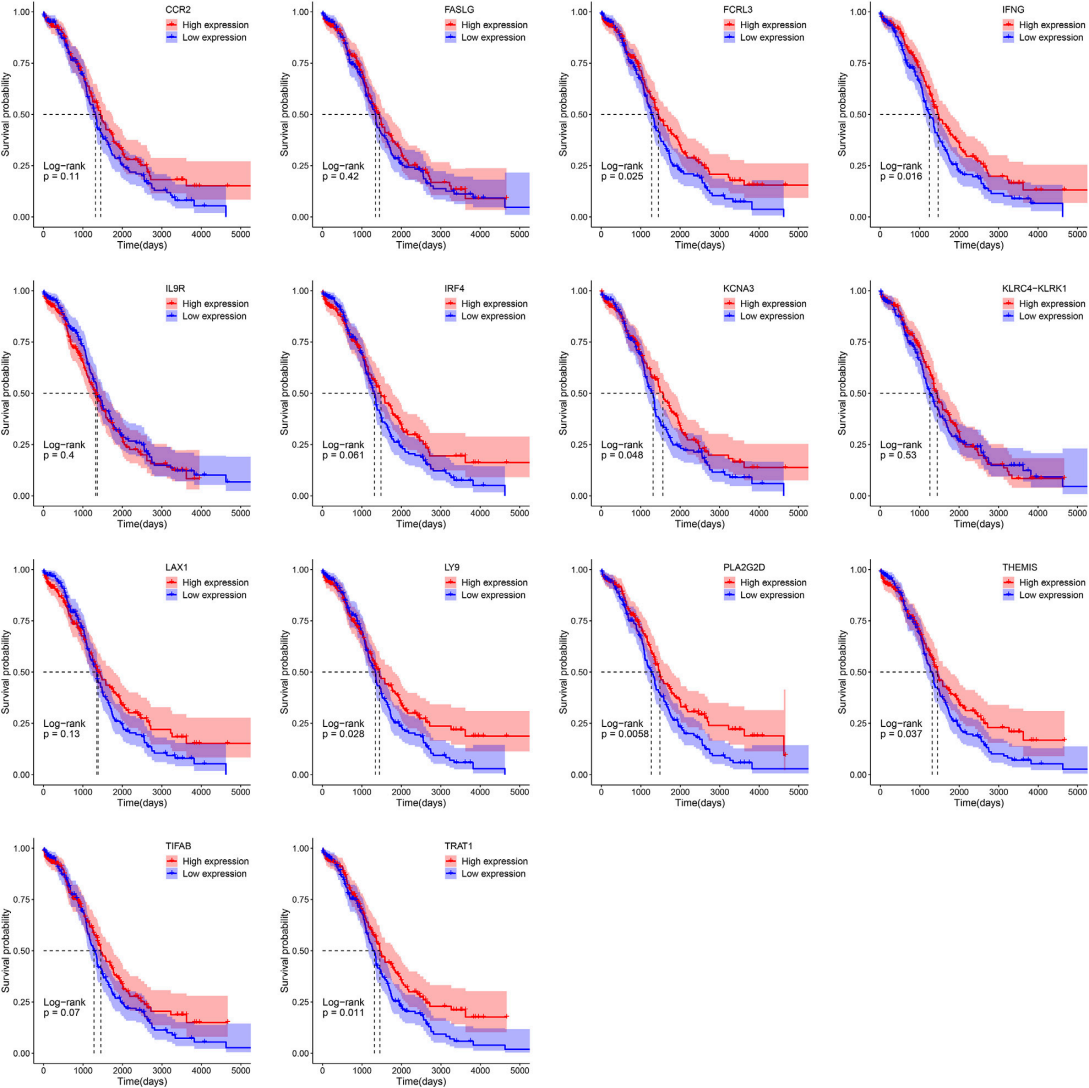
Survival analysis of 14 key genes in OV based on the Kaplan-Meier plotter.

Patients were separated into the low- and high-expression groups using the median gene expression level as cut-offs. Kaplan-Meier survival analysis showed that high expression group of FCRL3 (*p* = 0.025), IFNG (*p* = 0.016), KCNA3 (*p* = 0.048), LY9 (*p* = 0.028), PLA2G2D (*p* = 0.0058), THEMIS (*p* = 0.037), and TRAT1 (*p* = 0.011) displayed a better OS rate than the low expression group respectively (Figure 5).

### Correlations Between Key Genes and Immune Cells Infiltrated in OV

**FIGURE 6:**
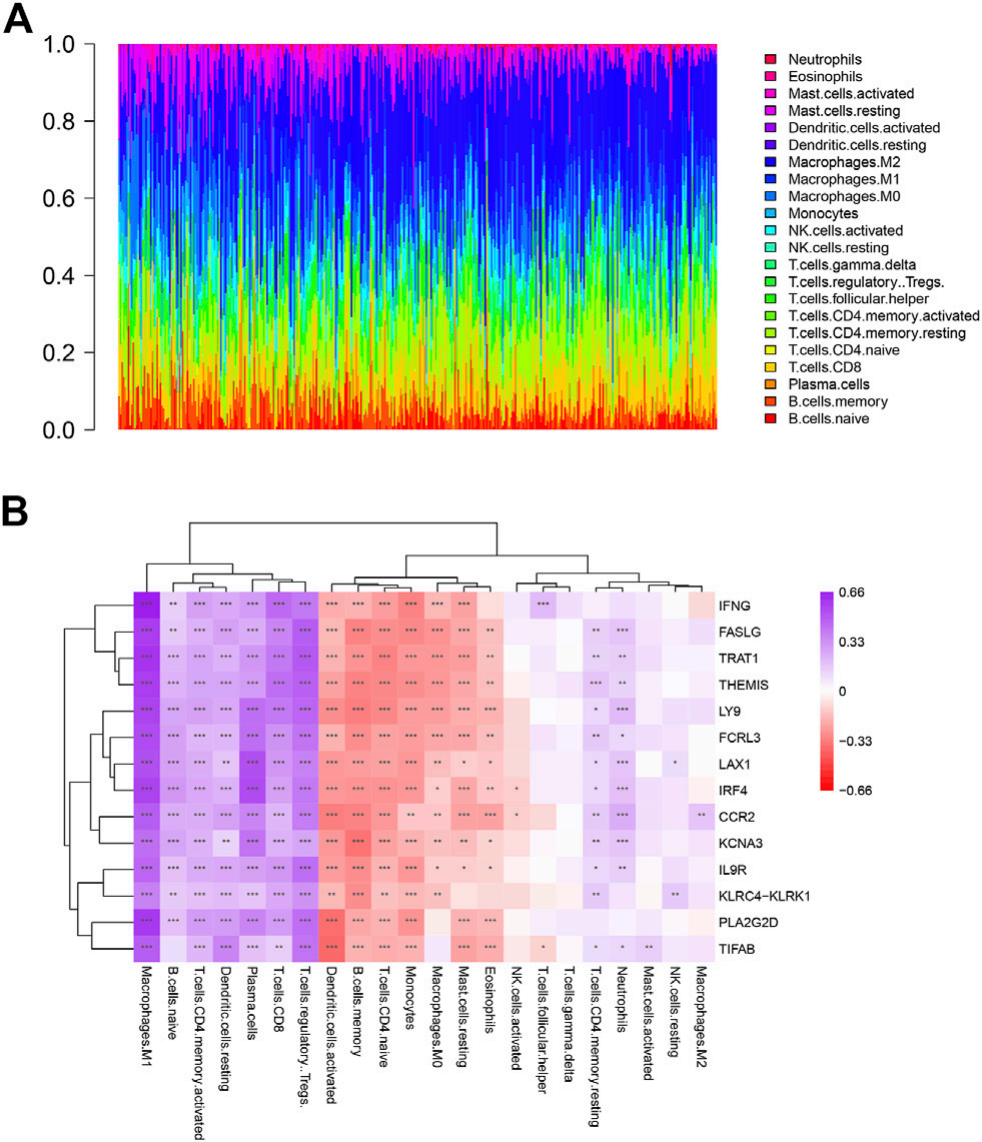
Profile of immune cells in OV and correlation analysis between genes and immune cells. **(A)** The proportion of 22 kinds of immune cells in ovarian tumor samples. **(B)** Correlation analysis between the expression of key genes and immune cells abundance.

To explore the immune microenvironment of OV, we used the CIBERSORT algorithm to estimate the relative abundance of 22 types of immune cells. As shown in Figure 6A, there existed various kinds of immune cells in each sample, the most abundant immune cells were macrophages. Additionally, the relation between the expression levels of key genes and immune cell type abundance was investigated (Figure 6B). Most of 14 key genes had a highly positive correlation with M1 macrophages, naive B cells, activated memory CD4 T cells, resting DCs (Dendritic Cells), CD8 T cells, plasma cells, Treg cells and neutrophils. On the contrary, most of key genes negatively correlated to activated DCs, memory B cells, naive CD4 T cells, monocytes, M0 macrophages, resting mast cells and eosinophils. In addition, IFNG was significantly positively correlated with Tfh cells. Therefore, it suggested that these 14 key genes could regulate above tumor-infiltrating immune cells of OV.

### The Relative Abundances of Immune Cells Associate With Prognosis

**FIGURE 7:**
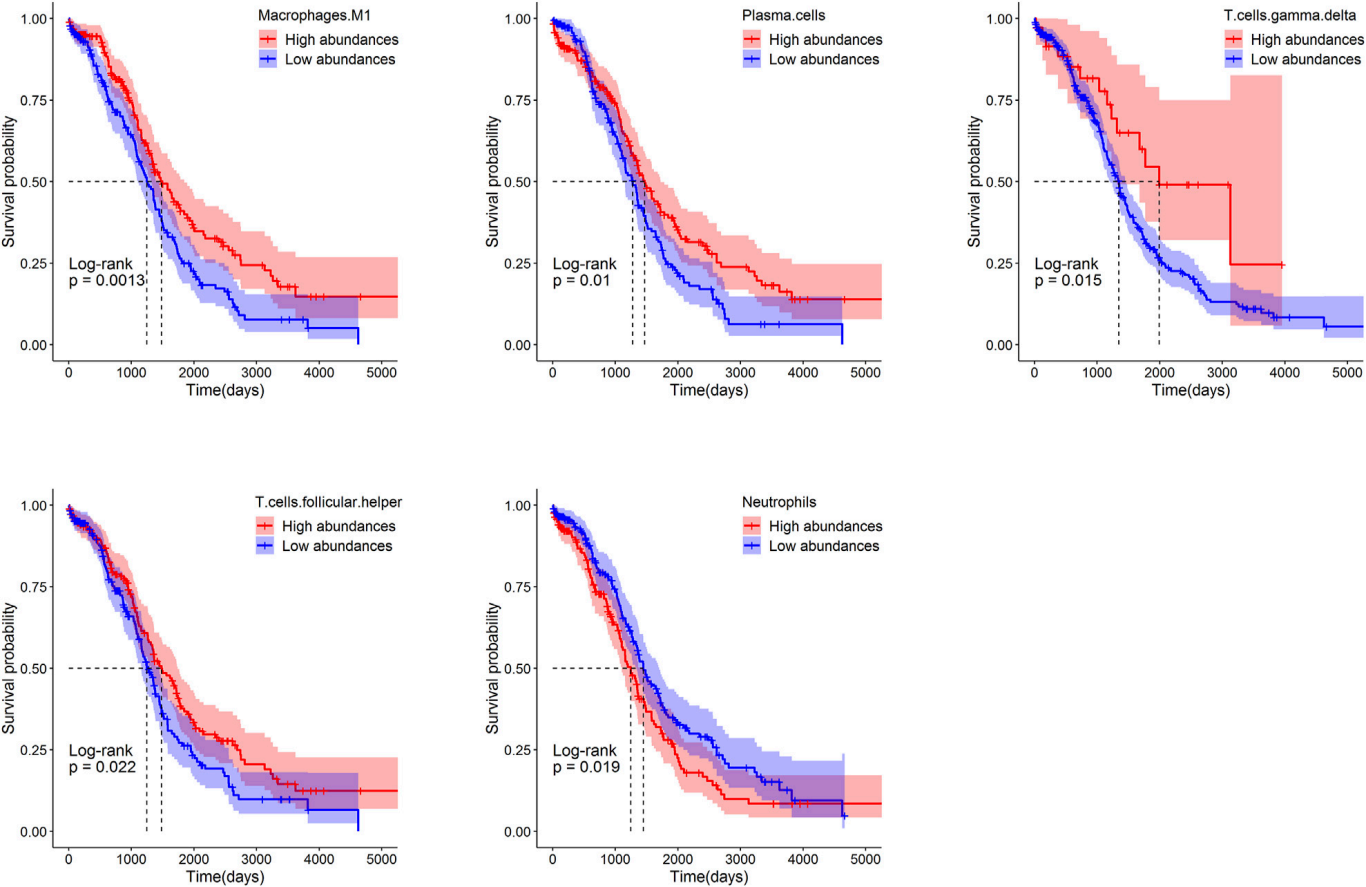
Survival analysis of immune cells in ovarian based on the Kaplan-Meier plotter.

Patients were separated into the low- and high-groups according to the median of relative abundances of immune cells. The higher relative abundances of immune cells of macrophages M1(*p* = 0.0013), plasma cells (*p* = 0.01), γδT (*p* = 0.015) and Tfh (*p* = 0.022) displayed a better OS rate than the lower relative abundances group. On the contrary, the lower relative abundances of immune cells of neutrophils (*p* = 0.019) displayed a better OS rate than the higher relative abundances group (Figure 7).

## Discussion

In this study, 14 immune-related key genes (IFNG, FASLG, TRAT1, THEMIS, LY9, FCRL3, LAX1, IRF4, CCR2, KCNA3, IL9R, KLRC4-KLRK1, PLA2G2D, and TIFAB) were screened out by WGCNA. Seven of 14 genes were significantly associated with the OS of OV. These key genes had potential regulatory effects on tissues infiltrating immune cells of ovarian cancer, especially M1 macrophages, Tregs, plasma cells.

Results showed that these 14 key genes were mainly enriched in T cell receptor activation and differentiation, immune receptor activation and binding, and NK cells mediated cytotoxicity. IFNG, FASLG, KLRC4-KLRK1 genes were enriched in T cell activation and differentiation and NK cell cytotoxicity simultaneously. IFNG is one of the main anti-tumor cytokines secreted by NK and T cells. FASLG, and KLRC4-KLRK1 are all involved in the apoptosis and cytotoxicity of NK and T cells (Alderson et al., 1995; Schneider et al., 1997; Tanaka et al., 1998). Other key genes enriched in T cell activation and differentiation were PLA2G2D, CCR2, LAX1, LY9, IRF4, and THEMIS. CCR2 and LY9 promote T-cell differentiation into a Th17 cell (Desai et al., 1991; Chatterjee et al., 2012; Sintes et al., 2013; Scott et al., 2015); IRF4 and THEMIS are critical genes for of CD8^+^ T cells differentiation and maintenance. PLA2G2D inhibit excessive Th1 immune response (Huber and Lohoff, 2014; Brzostek et al., 2020). LAX1 negatively regulates the signal pathway mediated by TCR (T cell receptor) (Zhu et al., 2002). Meanwhile, TRAT1 regulate the TCR expression and TCR-mediated signaling (Kirchgessner et al., 2001). The Kv1.3 potassium channel encoded by KCNA3 is involved in the calcium influx of T cells (Kang et al., 2016). IL9R drives γδT cells activation and promote CD8 T cell expansion (Guggino et al., 2016; Wang et al., 2020); FCRL3 promotes TLR9-induced B-cell proliferation, activation and survival but inhibits antibody production and suppresses plasma cell differentiation (Li et al., 2013). TIFAB have a role in the immune cell function (Niederkorn et al., 2020).

We analyzed the association between clinical characteristics (disease type, grade, stage and sample type) and the expression of key genes. As a result, we found KCNA3 was significantly related to disease stage. FCRL3, IFNG and TRAT1 were related to recurrence. What was more, a higher expression of each of these four genes and other three genes (LY9, PLA2G2D and THEMIS) showed a significantly superior OS than that of the lower expression. In addition, six of the above 7 key genes were higher expressed in OV tissues than those in normal control excepted KCNA3 (Figure 4). We proposed that the increased expression of these immune scores related genes may benefit the OV prognosis.

The correlation analysis between key genes and infiltrating immune cells showed that the main immune cell subtypes positively related to the key genes were immune effector cells (M1 macrophages and CD8 T cells), plasma cells with antibody secretion function, Treg cells and activated memory CD4 T cells. Immune cells negatively related to key genes include naive CD4 T cells and M0 macrophages, activated DC cells and memory B cells. In addition, IFNG is significantly positively correlated with Tfh cells, and most of the key genes are positively correlated with neutrophils. Results indicated that key genes up-regulated immune effector cells while improving immune regulatory functions, which may be to inhibit excessive immune responses. Furthermore, these key genes inhibited the undifferentiated state of macrophages and CD4 T cells, and promoted the development of plasma cells from B cells which activated by Tfh cells. However, the functions of these key genes in OV need further investigate by vitro and vivo experiments.

In addition, results of survival analysis showed that the higher abundances of immune effector cells (M1 macrophages and γδT cells) and cells related to antibody production (Tfh cells and plasma cells) predicted a better OV prognosis. It was reported that the increased ratio of M1/M2 is a beneficial factor for OS of OV (Zhang et al., 2014). However, the higher abundance of neutrophils indicates a poorer OV prognosis. It was reported that the presence of neutrophils and elevated neutrophil—lymphocyte ratio (NLR) at the time of initial diagnosis was a predictor for shorter survival in ovarian cancer patients (Komura et al., 2018). We speculated that this might be due to the rapid and severe inflammatory response caused by neutrophils. Taken together, both improved the immune response and immune regulation were necessary for a better OV prognosis.

In conclusion, we obtained 14 immune score-related key genes of OV. Seven of them were significantly related to the OV prognosis. These key genes may regulate the immune function of tumor-infiltrating immune cells. Enhanced immune responses and appropriate immune regulations may be beneficial to OV prognosis. However, the limitation of this article is that a larger sample size and clinical information are still needed, the molecular mechanism of these key genes in the prognosis of ovarian cancer and the regulatory mechanism of infiltrating immune cells need to be confirmed by further basic experiments. Our results provide potential prognostic markers and new perspectives on immunotherapy for OV.

## Data Availability

The datasets presented in this study can be found in online repositories. The names of the repository/repositories and accession number(s) can be found in the article/Supplementary Material.
