# Supplementary material for: Identification of Immune-Related Key Genes in Ovarian Cancer Based on WGCNA
Source: Front Genet. 2021 Nov 15;12:760225. doi: 10.3389/fgene.2021.760225 (PMC8634599; doi:10.3389/fgene.2021.760225)
Supplement: Supplementary file 2 [file Table3.DOCX]

**ESTIMATE**

library("estimate")

load("ex10.Rdata")

filterCommonGenes(input.f="ex10.txt",

output.f="commonGenes.gct",

id="GeneSymbol")

estimateScore("commonGenes.gct", "estimatescore.gct", platform="affymetrix")

scores=read.table("estimatescore.gct",skip = 2,header = T)

rownames(scores)=scores[,1]

scores=t(scores[,3:ncol(scores)])

scores

scores2<-as.data.frame(scores)

write.csv(scores2,"scores2.csv",row.names =T)

**WGCNA**

library(WGCNA)

library(data.table)

library(stringr)

library(openxlsx)

allowWGCNAThreads()

ALLOW_WGCNA_THREADS=4

memory.limit(size = 20000)

load("data2.Rdata")

exprSize = checkSets(data2,checkStructure = T)

gsg = goodSamplesGenes(data2)

gsg$allOK

if (!gsg$allOK) {

data2 = data2[gsg$goodSamples, gsg$goodGenes]

}

gsg = goodSamplesGenes(data2)

gsg$allOK

#

powers = c(c(1:10), seq(from = 12, to=20, by=2))

sft = pickSoftThreshold(data2, powerVector = powers, verbose = 5)

pdf("001SFT.pdf",width=10,height = 6)

par(mfrow = c(1,2));

cex1 = 0.9;

plot(sft$fitIndices[,1], -sign(sft$fitIndices[,3])*sft$fitIndices[,2],

xlab="Soft Threshold (power)",ylab="Scale Free Topology Model Fit,signed R^2",type="n",

main = paste("Scale independence"));

text(sft$fitIndices[,1], -sign(sft$fitIndices[,3])*sft$fitIndices[,2],

labels=powers,cex=cex1,col="red");

abline(h=0.90,col="red")

plot(sft$fitIndices[,1], sft$fitIndices[,5],

xlab="Soft Threshold (power)",ylab="Mean Connectivity", type="n",

main = paste("Mean connectivity"))

text(sft$fitIndices[,1], sft$fitIndices[,5], labels=powers, cex=cex1,col="red")

dev.off()

#

softPower <- sft$powerEstimate

softPower

adjacency = adjacency(data2, power = softPower);

TOM = TOMsimilarity(adjacency)

dissTOM = 1-TOM

hierTOM = hclust(as.dist(dissTOM),method="average")

k <- softConnectivity(datE=data2,power=softPower)

pdf("002K.pdf",width=10,height = 6)

par(mfrow = c(1,2))

hist(k)

scaleFreePlot(k,main="Check Scale free topology\n")

dev.off()

geneTree = hclust(as.dist(dissTOM), method = "average");

pdf("003Tree.pdf",width=10,height = 6)

plot(geneTree, xlab="", sub="", main = "Gene clustering on TOM-based dissimilarity",

labels = FALSE, hang = 0.04);

minModuleSize = 50;

dynamicMods = cutreeDynamic(dendro = geneTree, distM = dissTOM,

deepSplit = 2, pamRespectsDendro = FALSE,

minClusterSize = minModuleSize);

table(dynamicMods)

#

dynamicColors <- labels2colors(dynamicMods)

table(dynamicColors)

plotDendroAndColors(geneTree, dynamicColors, 'Dynamic Tree Cut',

dendroLabels = FALSE, addGuide = TRUE, hang = 0.03, guideHang = 0.05,

main = 'Gene dendrogram and module colors')

dev.off()

MEList = moduleEigengenes(data2, colors = dynamicColors)

MEs = MEList$eigengenes

MEDiss = 1-cor(MEs);

METree = hclust(as.dist(MEDiss), method = "average")

pdf("005MEs.pdf",width=5,height = 4)

plotEigengeneNetworks(MEs,

"Eigengene adjacency heatmap",

marHeatmap = c(3,4,2,2),

plotDendrograms = FALSE,

xLabelsAngle = 90)

dev.off()

pdf("006Clustering.pdf",width=5,height = 4)

plot(METree,

main = "Clustering of module eigengenes",

xlab = "",

sub = "")

MEDissThres = 0.2

abline(h=MEDissThres, col = "red")

dev.off()

merge_modules = mergeCloseModules(data2, dynamicColors, cutHeight = MEDissThres, verbose = 3)

mergedColors = merge_modules$colors;

mergedMEs = merge_modules$newMEs;

pdf("007mergedTree.pdf",width=10,height = 6)

plotDendroAndColors(geneTree, cbind(dynamicColors, mergedColors),

c("Dynamic Tree Cut", "Merged dynamic"),

dendroLabels = FALSE, hang = 0.03,

addGuide = TRUE, guideHang = 0.05)

dev.off()

#

load("pho6.Rdata")

#

#

pdf("015Intramodular Connectivity and Module Membership01.pdf",width=5,height = 8)

moduleTraitCor_noFP <- cor(mergedMEs, pho6[,2], use = "p");

moduleTraitPvalue_noFP = corPvalueStudent(moduleTraitCor_noFP, nSamples);

textMatrix_noFP <- paste(signif(moduleTraitCor_noFP, 2), "\n(", signif(moduleTraitPvalue_noFP, 1), ")", sep = "");

par(mar = c(6, 10, 3, 3));

labeledHeatmap(Matrix = moduleTraitCor_noFP,

xLabels = names(pho6)[2],

yLabels = names(mergedMEs),

ySymbols = names(mergedMEs),

colorLabels = FALSE,

colors = blueWhiteRed(50),

textMatrix = textMatrix_noFP,

setStdMargins = FALSE,

cex.text = 0.65,

zlim = c(-1,1),

main = paste("Module-trait relationships"))

dev.off()

#

cor_ADR <- signif(WGCNA::cor(pho6,mergedMEs,use="p",method="pearson"),5)

p.values <- corPvalueStudent(cor_ADR,nSamples=nrow(pho6))

#

GS1 <- as.numeric(WGCNA::cor(pho6[,"ImmuneScore"],data2,use="p",method="pearson"))

GeneSignificance <- abs(GS1)

ModuleSignificance <- tapply(GeneSignificance,mergedColors,mean,na.rm=T)

ModuleSignificance

which.max(ModuleSignificance[names(ModuleSignificance)!="grey"])

maxmodule<-which.max(ModuleSignificance[names(ModuleSignificance)!="grey"])

maxmodule1<-as.data.frame(maxmodule)

scolor<-rownames(maxmodule1)

kDiff=kIn-kOut=2*kIN-kTotal

Alldegrees1=intramodularConnectivity(ADJ1, moduleColors)

ImmuneScore = as.data.frame(pho6$ImmuneScore);

names(ImmuneScore) = "ImmuneScore"

# Add the weight to existing module eigengenes

MET = orderMEs(cbind(mergedMEs, ImmuneScore))

# Plot the relationships among the eigengenes and the trait

pdf("017ImmuneScore.pdf",width=3,height = 4);#下、左、上、右

par(cex = 0.55)

plotEigengeneNetworks(MET, "", marDendro = c(0,7,1,2), marHeatmap = c(6,7,1,2), cex.lab = 0.8, xLabelsAngle = 90)

dev.off()

#

corType="pearson"

if (corType=="pearson") {

geneModuleMembership = as.data.frame(cor(data2, mergedMEs, use = "p"))

MMPvalue = as.data.frame(corPvalueStudent(

as.matrix(geneModuleMembership), nSamples))

} else {

geneModuleMembershipA = bicorAndPvalue(data2, mergedMEs, robustY=ifelse(corType=="pearson",T,F))

geneModuleMembership = geneModuleMembershipA$bicor

MMPvalue = geneModuleMembershipA$p

}

if (corType=="pearson") {

geneTraitCor = as.data.frame(cor(data2, pho6, use = "p"))

geneTraitP = as.data.frame(corPvalueStudent(

as.matrix(geneTraitCor), nSamples))

} else {

geneTraitCorA = bicorAndPvalue(data2, pho6, robustY=ifelse(corType=="pearson",T,F))

geneTraitCor = as.data.frame(geneTraitCorA$bicor)

geneTraitP = as.data.frame(geneTraitCorA$p)

}

module = scolor

pheno = "ImmuneScore"

modNames = substring(colnames(mergedMEs), 3)

module_column = match(module, modNames)

pheno_column = match(pheno,colnames(pho6))

moduleGenes = moduleColors == module

pdf("019MMvsGS.pdf",width=5,height = 5)

par(mfrow = c(1,1))

verboseScatterplot(abs(geneModuleMembership[moduleGenes, module_column]),

abs(geneTraitCor[moduleGenes, pheno_column]),

xlab = paste("Module Membership in", module, "module"),

ylab = paste("Gene significance for", pheno),

main = paste("Module membership vs. gene significance\n"),

cex.main = 1.2, cex.lab = 1.2, cex.axis = 1.2, col = module)

abline(h=0.5, col = "red")

abline(v=0.7, col = "red")

dev.off()

#

wsc<-which(str_detect(colnames(datKME),scolor))

head(data2[1:4,1:4])

class(data2)

datKME=signedKME(data2, mergedMEs, outputColumnName="MM.")

FilterGenes= which(mergedColors==scolor)[abs(GS1)[which(mergedColors==scolor)]> 0.5 & abs(datKME[moduleGenes,wsc])>0.7]

which(FilterGenes=="TRUE")

dimnames(data.frame(data2))[[2]][FilterGenes]

trait_hubGenes<-colnames(data2)[FilterGenes]

write.csv(trait_hubGenes,"hubgene immune.csv")

**clusterProfiler**

library(ggplot2)

library(clusterProfiler)

library(org.Hs.eg.db)

library(AnnotationDbi)

library(stats4)

library(BiocGenerics)

library(parallel)

#

load('hubgene.Rdata')

df <- bitr(unique(hubgene$gene_name), fromType = "SYMBOL",

toType = c( "ENTREZID"),

OrgDb = org.Hs.eg.db)

gene_diff<-df$ENTREZID

#

kk <- enrichKEGG(gene = gene_diff,

organism = 'hsa',

pvalueCutoff = 0.05,

qvalueCutoff = 0.05)

barKK<-barplot(kk,drop=T,showCategory = 10,title='bar_KEGG')

ggsave(file="barKKDIFF.png",plot=barKK,width=8,height = 4.6)

write.csv(summary(kk),"kk.diff.csv",row.names =F)

#

ego_CC <- enrichGO(gene = gene_diff,

OrgDb= org.Hs.eg.db,

ont = "CC",

pAdjustMethod = "BH",

readable = T)

ego_BP <- enrichGO(gene = gene_diff,

OrgDb= org.Hs.eg.db,

ont = "BP",

pAdjustMethod = "BH",

readable = T)

ego_MF <- enrichGO(gene = gene_diff,

OrgDb= org.Hs.eg.db,

ont = "MF",

pAdjustMethod = "BH",

readable = T)

barcc<-barplot(ego_CC, showCategory=5,title="bar_CC")

ggsave(file="barcc.png",plot=barcc,width=7.54,height = 2)

barBP<-barplot(ego_BP, showCategory=5,title="bar_BP")

ggsave(file="barBP.png",plot=barBP,width=7.54,height = 2.3)

barMF<-barplot(ego_MF, showCategory=5,title="bar_MF")

ggsave(file="barMF.png",plot=barMF,width=7.51,height = 2.3)

write.csv(summary(ego_MF),"ego_MF.csv",row.names =F)

write.csv(summary(ego_BP),"ego_BP.csv",row.names =F)

write.csv(summary(ego_CC),"ego_CC.csv",row.names =F)

write.csv(df,"df.csv",row.names =F)

**Corrplot calculations**

library(PerformanceAnalytics)

library(xts)

library(zoo)

library(corrplot)

load('TSratio.Rdata')

a<-TSratio[,-c(1,2,3,18,41,42,43)]

colnames(a)

exoasratio <- rcorr(as.matrix(a),type = "spearman")

write.csv(exoasratio$r,"exoasratior.csv")

write.csv(exoasratio$n,"exoasration.csv")

write.csv(exoasratio$P,"exoasratiop.csv")

**Kaplan-Meier survival analysis**

library(dplyr)

library(survival)

library(survminer)

library(ggplot2)

library(ggpubr)

load('TS.Rdata')

a<-TS

#

group<-ifelse(a$IFNG>median(a$IFNG),'High expression','Low expression')

table(group)

fit<-survfit(Surv(OS.time,OS)~group,data=a)

gg<-ggsurvplot(fit,risk.table=F,

conf.int=TRUE,

surv.median.line = 'hv',

palette = c("red","blue"),

pval=TRUE,#log-rank

pval.size=5,

legend = c(0.8,0.9),#

legend.title='IFNG',#

legend.labs = c("High expression","Low expression"),

xlab="Time(days)",

font.legend =13,

pval.coord= c(0,0.2),

pval.method.coord= c(0,0.25),

pval.method=TRUE)#

ggsave("sur gene IFNG .png",plot = print(gg),width = 5,height = 5,units = "in")

#
